# The Influence of Genome Composition and Temperature on the Hatching Success and Development of the Offspring of Allotriploid *Cobitis* (Pisces: Cobitidae) Females

**DOI:** 10.3390/ijms262110669

**Published:** 2025-11-01

**Authors:** Sara Duda, Olga Jablonska, Alicja Boroń, Roman Kujawa, Karel Janko, Dorota Juchno

**Affiliations:** 1 Department of Zoology, Faculty of Biology and Biotechnology, University of Warmia and Mazury in Olsztyn, 10-719 Olsztyn, Poland; sara.felinska@student.uwm.edu.pl (S.D.); olga.jablonska@uwm.edu.pl (O.J.); alibo@uwm.edu.pl (A.B.); 2 Department of Ichthyology and Aquaculture, University of Warmia and Mazury in Olsztyn, 10-720 Olsztyn, Poland; reofish@uwm.edu.pl; 3 Laboratory of Non-Mendelian Evolution, Institute of Animal Physiology and Genetics, Czech Academy of Sciences, Rumburska 89, 27721 Libechov, Czech Republic; janko@iapg.cas.cz; 4 Department of Biology and Ecology, Faculty of Natural Sciences, University of Ostrava, Chittussiho 10, 71000 Ostrava, Czech Republic

**Keywords:** loaches, hybridization, polyploidization, genomic identification, larvae development, larvae abnormalities, body length

## Abstract

Hybridization and polyploidization are key evolutionary forces shaping fish biodiversity. But their interaction with environmental factors, such as temperature, remains poorly understood. This study examined how maternal genome composition and incubation water temperature influence the hatching success, ploidy structure, morphology and early growth of polyploid *Cobitis* larvae. Crosses were performed using triploid *Cobitis* females with three genomic compositions (EEN, EET and ETN), representing combinations of genomes from *C. elongatoides* (E), *C. taenia* (T) and *C. tanaitica* (N), and diploid *C. taenia* males as sperm donors. Fertilized eggs were incubated at 18 °C, 22 °C and 28 °C. Triploid and tetraploid offspring occurred in comparable proportions on average across all groups, but developmental abnormalities were significantly more observed in tetraploid larvae. Females with EET and ETN genomes achieved higher hatching success than those with the EEN genome. Temperature had a pronounced effect on developmental timing and success: hatching occurred earliest at 28 °C, but survival decreased and abnormalities were most frequent. These results highlight genome- and temperature-dependent trade-offs in early development of *Cobitis* hybrids, providing new insight into reproductive dynamics and the potential resilience of polyploid systems under climate warming.

## 1. Introduction

In poikilothermic animals such as teleost fish, ambient temperature modulates growth, development, and reproduction by acting on multiple levels of the brain–pituitary–gonad axis [1,2]. Warmer-than-optimal temperatures can affect every stage of reproductive development, from gametogenesis to spawning, altering both timing and success rates [1]. Experimental studies across diverse taxa, including Atlantic salmon *Salmo salar* [3], rainbow trout *Oncorhynchus mykiss* [4], European catfish *Silurus glanis* [5], Nile tilapia *Oreochromis niloticus* [6] and aquarium species such as spotted scat *Scatophagus argus* [7] and Korean rockfish *Sebastes schlegelii* [8] consistently demonstrate that temperature critically influences gonad maturation, fertilization, and embryonic survival.

Polyploidy, the presence of more than two chromosome sets, is a major evolutionary event that occurred repeatedly in both plants and animals. Polyploidization has played a pivotal role in vertebrate evolution. Ancient whole-genome duplications (WGDs), likely of autopolyploid origin, were fundamental to early vertebrate diversification [9,10,11]. In contrast, recent polyploidization events, particularly those of allopolyploid origin, are rare in vertebrates and have been described mainly in fish and amphibians [12,13]. The genus *Cobitis*, which includes naturally occurring hybrids with different ploidy levels, therefore provides an important model for studying the mechanisms and consequences of hybridization and polyploidization processes [14,15,16,17]. These small benthic fishes, usually about 10 cm in length, comprise nearly 110 species distributed across Eurasia and North Africa [18], including 33 species in Europe. At least three of these species occur in Central Europe—the spined loach (*C. taenia*), the Danubian loach (*C. elongatoides*, EE) and *C. tanaitica*, NN—which form complexes of natural hybrids that include numerous polyploid lineages [19]. Triploid (3n) *Cobitis* females carry different genomic combinations depending on their parental species. Within the European range, the most common genomic compositions are EEN, EET, ETN, ETT and ENN [19]. In Poland, mixed diploid—polyploid populations consist mainly of diploid (2n) hybrids (ET) and 3n hybrid females with the EEN, EET, ETN and ETT genomes, whereas allotetraploids (4n) of both sexes rarely exceed [20,21,22]. Although *C. tanaitica* has not been found in Polish waters, its genome persists in 3n and 4n hybrids [19,20]. These hybrid forms are morphologically similar and can be distinguished only through cytogenetic or molecular analyses [23]. The origin of these hybrids is associated with postglacial dispersal and secondary contact between previously isolated populations derived from Ponto-Caspian refugia. Following the retreat of the glaciers, these populations recolonized Central and Eastern Europe, where contact zones among *C. taenia*, *C. elongatoides*, and *C. tanaitica* were established, promoting the formation of hybrid and clonal lineages. Mito-nuclear discordance, resulting from historical hybridization and mitochondrial capture, explains the presence of *C. tanaitica* mitochondrial haplotypes within *Cobitis* populations dominated by other nuclear genomes [24].

Reproductive biology in *Cobitis* is equally complex. These fishes spawn in batches, from May to July [25]. Diploid and triploid hybrid males are typically sterile, whereas females reproduce clonally through gynogenesis or, less frequently, sexually to produce 4n offspring of both sexes [22,26,27]. Gynogenetic reproduction is triggered by sperm activation without genetic contribution, and it is supported by premeiotic genome endoreplication, which restores diploidy in clonal oocytes before meiosis [28]. Unlike triploids, tetraploid females are rarely fertile and produce fewer eggs than diploids and triploids [25]. In our previous study, 4n progeny obtained from induced spawning experiments showed markedly lower survival than clonal 3n ones during early development [29]. In that study, Juchno et al. [29] demonstrated that the initial proportions of 3n and 4n individuals hatching were comparable; however, the number of tetraploids decreased significantly over time, reaching levels similar to those found in the environment. Furthermore, some polyploid offspring exhibited various developmental deformities during embryogenesis and larval stages [29].

Fish of the genus *Cobitis* thus provide a unique model for studying also how environmental factors, particularly water temperature, influence reproductive outcomes. Polyploid organisms are known for their ability to tolerate environmental fluctuations, and in some taxa polyploidy has been linked to enhanced resilience to thermal stress. For example, certain polyploid plants show increased cold tolerance [30], or benefit from post–fire warming [31]. Moreover, 3n hybrid *Pelophylax* frogs exhibit faster growth and greater body mass than diploids at lower temperatures [32]. Triploid *Cobitis* hybrids possess larger nuclei and cells, as well as a significantly lower metabolic rate than diploids [33], which may influence their developmental response to temperature. In the context of ongoing climate warming [34], understanding how water incubation temperature influences reproduction in polyploid *Cobitis* is essential for predicting the persistence of their lineages, which spawn across a broad thermal range 18–28 °C [35]. Despite extensive research on fish reproduction under varying thermal regimes, the effects of temperature and genome composition in natural hybrid polyploid systems remain poorly understood. To our knowledge, no previous study has compared hatching success, ploidy structure, and early larval development among allotriploid *Cobitis* females with known genomic composition under controlled temperature regimes (18 °C, 22 °C, and 28 °C).

The present study aimed to determine how maternal genomic composition and incubation temperature affect reproductive success, developmental stability, and early larval growth in polyploid *Cobitis* hybrids. We hypothesized that (1) developmental success, including hatching rate, frequency of abnormalities, and larval body length, differs among 3n females with distinct genomic constitutions (EEN, EET, ETN) (2) incubation temperature affects both hatching success and offspring phenotype, and (3) the proportion of gynogenetic (3n) and sexually derived (4n) progeny remains similar during the first few days after hatching across temperatures but may vary among genomic lineages depending on their reproductive mode preference.

## 2. Results

### 2.1. Female Genome Structure

Based on molecular and karyotype analyses, the triploid parental females were classified according to their inferred parental subgenomes: *C. taenia* (T), *C. elongatoides* (E) and *C. tanaitica* (N). These were assigned to three genomic groups: EEN, EET and ETN. These groups possess the following numbers of chromosomes: 3n = 75, 3n = 74* and 3n = 74, respectively. Of the 20 triploid parental females analyzed, 5 exhibited an EEN composition, 5 an EET composition and 10 an ETN composition.

### 2.2. Hatching Success

Overall, none of the tested temperatures precluded offspring production. Furthermore, a significant effect of temperature and genomic composition on developmental success has been demonstrated. The duration of embryonic development varied; larvae incubated at 28 °C hatched after three days, those incubated at 22 °C hatched after four days, and those incubated at 18 °C hatched after five days post-fertilization. Table 1 shows the number of eggs, the number of larvae hatched, and the resulting hatching success rates for each family across the three 3n female genomes: EEN, EET and ETN—at three experimental temperatures: 18 °C, 22 °C and 28 °C. Regardless of genome composition, the mean hatching success rates were 32.9%, 31.7%, and 14.8% at 18 °C, 22 °C, and 28 °C, respectively. However, the mean hatching success of offspring was 10.3% for the EEN genome, 31.7% for the EET genome and 31.9% for the ETN genome, regardless of water temperature.

Offspring of EEN females generally had low hatching success. Among offspring derived from ETN females, hatching success was 16.8% at 18 °C, followed by 7.7% at 22 °C and 6.4% at 28 °C. No significant differences were found within the EEN genome at different temperatures (*p* > 0.05). However, we emphasize that this lack of difference may be caused by interfamilial differences where several families (F35M35, F37M37, F39M39 and F47M47) indeed showed no hatching in one or two temperature groups, whereas one family, F35M35, showed consistently high success across temperatures, particularly at 18 °C (39.5%).

In the EET group, the mean hatching success was 31.2% at 18 °C, 49.8% at 22 °C, and 14.2% at 28 °C. Statistically significant differences were found between hatching success at 22 °C and 28 °C (*p* < 0.05). The F72M72 family stands out in this group, showing high efficiency at all temperatures (up to 84.5% at 18 °C).

Among offspring derived from ETN females, hatching success was 41.8% at 18 °C, 34.6% at 22 °C, and 19.3% at 28 °C. However, there were no statistically significant differences between the three temperatures (*p* > 0.05). Some ETN families (e.g., F78M78) exhibited very high hatching success at all temperatures. Others (e.g., F5M5 and F10M10) showed complete hatching failure at the highest temperature only, while some (e.g., F12M12) exhibited no hatching at 18 °C and 28 °C.

At temperatures of 18 °C and 28 °C, there were no statistically significant differences in hatching success between the studied genomes (*p* > 0.05). However, at 22 °C, EEN females exhibited significantly lower hatching success compared to EET and ETN females (*p* < 0.05).

**Figure 1:**
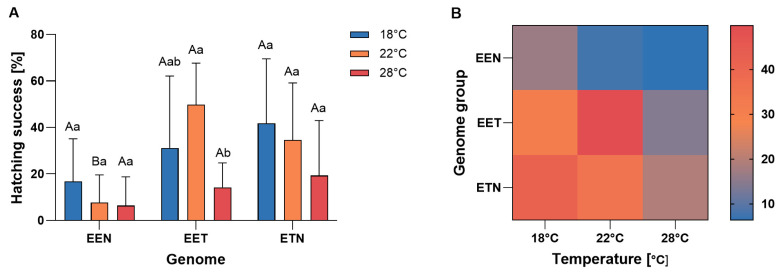
(**A**) Mean frequency of hatching success (%) of *Cobitis* progeny according to female genome composition (EEN, EET, and ETN) and incubation water temperature (18 °C, 22 °C or 28 °C). Bars represent mean ± SD. Statistical significance was determined by one-way ANOVA followed by Tukey’s post hoc test (*p*  <  0.05). Different uppercase letters indicate significant differences (*p* < 0.05) among genotypes within a given temperature, and different lowercase letters indicate significant differences (*p* < 0.05) among temperatures within each genotype (**B**) Heatmap illustrates hatching success (%) across genotypes (EEN, EET, ETN) and incubation water temperature (18 °C, 22 °C and 28 °C). Color intensity represents mean hatching success for each genome × temperature combination.

Interaction between maternal genome composition and incubation temperature was observed in terms of hatching success (Figure 1). Offspring of ETN females exhibited higher hatching success at 18 °C, whereas offspring of EET females demonstrated higher hatching success at 22 °C. In contrast, EEN offspring exhibited significantly reduced hatching success at 22 °C and 28 °C, achieving minimal success at higher temperatures. Overall, ETN and EET females produced offspring with a similar mean hatching success rate, while EEN females exhibited the lowest rate, particularly at the highest temperature (Figure 1).

### 2.3. Ploidy and Phenotype of Progeny

The ploidy status of randomly selected progeny during the three days post hatching revealed triploids (3n) and tetraploids (4n), as well as progeny at other ploidy levels which are not included in this study. Comparable proportions of triploid and tetraploid offspring were observed across all genome compositions (EEN, EET and ETN) and water temperatures tested (18 °C, 22 °C and 28 °C), although considerable variability was observed among individual crosses. Moreover, no consistent differences were observed between groups. No statistically significant differences were detected (*p* > 0.05; Table 2).

**Figure 2:**
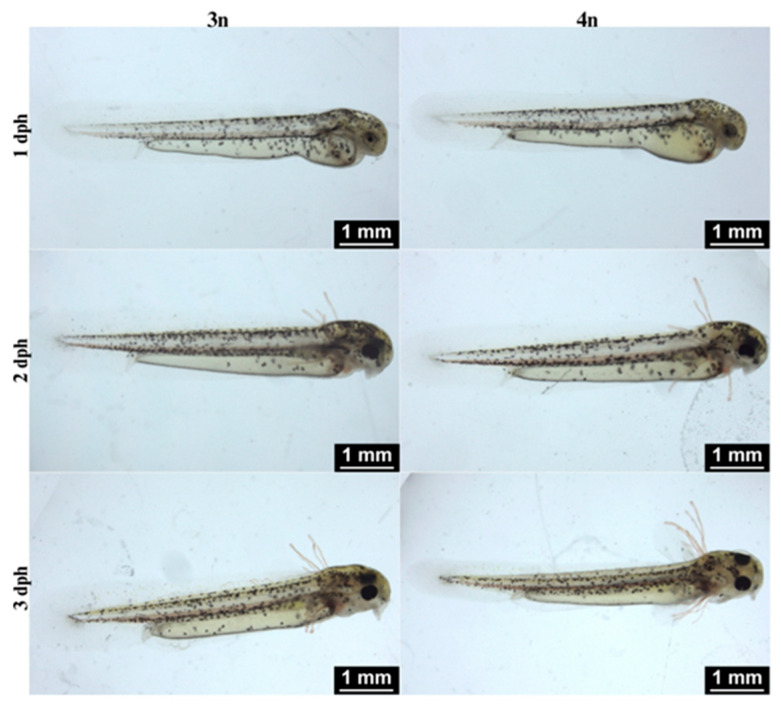
Properly developed triploid (3n) and tetraploid (4n) *Cobitis* larvae during the first three days post hatching. Early post-hatch development proceeded similarly in both ploidy levels, showing comparable timing of yolk sac absorption, fin fold formation, and pigmentation.

The developmental patterns of triploid and tetraploid offspring were highly similar (Figure 2). At 1 dph, numerous melanophores appeared across the larval body, including around the eyes. Blood circulation, the intestine and an elongated yolk sac were clearly visible, and a continuous fin fold had formed, connecting the dorsal, caudal and pelvic regions. By 2 dph, four pairs of filamentous external gills and small pectoral fins were present; the eyes had darkened, and the yolk sac had become smaller. By 3 dph, the external gills were still visible and the mouth had opened, while the pectoral fins had become more prominent and enlarged (Figure 2).

**Figure 3:**
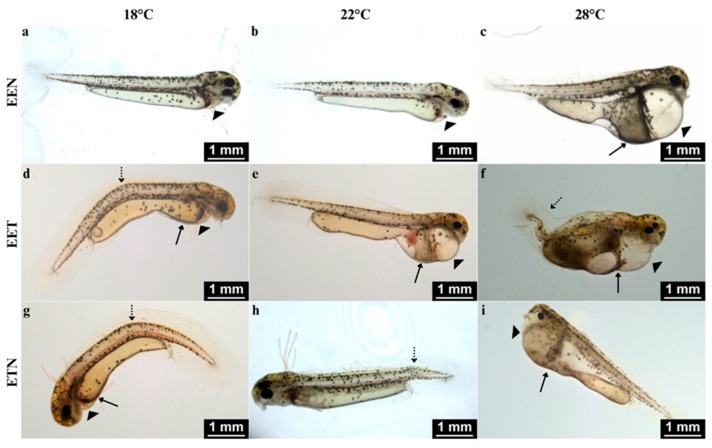
Examples of external morphological abnormalities in tetraploid (4n) *Cobitis* progeny of females with different genomic compositions: EEN (**a**–**c**), EET (**d**–**f**), and ETN (**g**–**i**). The most frequent malformations included cardiac edema, yolk sac edema, and axial deformities such as kyphosis, lordosis, and C-shaped body curvature. Yolk sac malformations often appeared as anterior enlargement, while some larvae also exhibited abnormal development of the external gills. Marks: arrowhead—cardiac edema; arrow yolk sac edema; dotted arrow—kyphosis, lordosis and axial curvature.

Physical deformities, which sometimes occurred in high numbers, were detected in both triploid and tetraploid offspring. A comparison of the percentage of abnormalities in 3n and 4n individuals, regardless of genome composition or water temperature, revealed a statistically significant difference: 43.0% of 4n offspring had abnormalities, compared to 25.2% of 3n offspring (*p* < 0.05). The most frequent abnormalities affected the heart cavity, yolk sac and body axis. Specifically, axial deformities such as kyphosis and lordosis, as well as lateral deformities including scoliosis and C-shaped curvature, were observed. Numerous oedemas developed within the heart cavity. Yolk sac malformations most often manifested as enlargement of the anterior region, giving it a pear-shaped appearance. Additionally, some larvae exhibited abnormally developed external gills (Figure 3).

**Figure 4:**
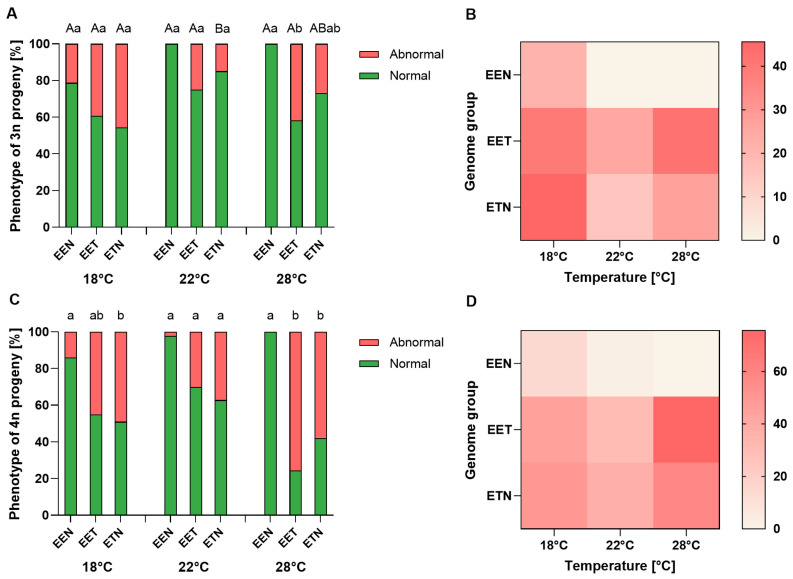
(**A**,**C**) Proportion of normal and abnormal larvae in triploid (3n; (**A**)) and tetraploid (4n; (**C**)) *Cobitis* progeny according to female genome composition (EEN, EET or ETN) and incubation water temperature (18 °C, 22 °C or 28 °C). Bars represent mean ± SD. Statistical significance was determined by one-way ANOVA followed by Tukey’s post hoc test (*p*  <  0.05). Different lowercase letters indicate significant differences (*p* < 0.05) in the percentage of abnormal larvae between genotypes within a given temperature. Different uppercase letters indicate significant differences (*p* < 0.05) in the percentage of abnormal larvae among individuals between temperatures (18 °C, 22 °C and 28 °C) within each genotype. (**B**,**D**) Heatmaps showing the frequency of abnormalities in triploid (3n; (**B**)) and tetraploid (4n; (**D**)) progeny across genotypes and incubation temperatures. Color intensity represents the mean frequency of abnormalities for each genome x temperature combination.

**Figure 5:**
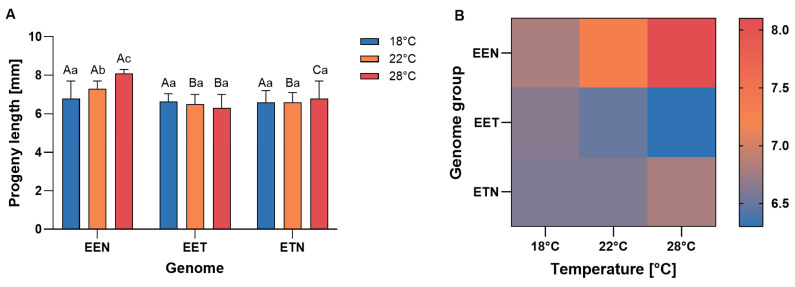
(**A**) Standard body length (mean ± SE) of *Cobitis* progeny (SL) according to female genome composition (EEN, EET and ETN) and incubation water temperature (18 °C, 22 °C and 28 °C). Different lowercase letters indicate significant differences (*p* ≤ 0.05) among temperatures within a given genome, and different uppercase letters indicate significant differences (*p* ≤ 0.05) among genomes (EEN, EET and ETN) within a given temperature. (**B**) Heatmap summarizing mean body length of *Cobitis* progeny according to maternal genome composition (EEN, EET, ETN) and incubation water temperature (18 °C, 22 °C and 28 °C). Color intensity represents the mean value for each genome x temperature combination.

Significant differences in the frequency of abnormalities were observed among triploid progeny in relation to the genome composition of the triploid female and the water temperature (see Figure 4). A significantly higher proportion of abnormal progeny were observed in females with the EET genome (41.7%) than in those with the EEN genome (0%) at a temperature of 28 °C (*p* < 0.05). More abnormalities were also observed at 18 °C (45.6%) than at 22 °C (15%) within the ETN genome (*p* < 0.05) (Figure 4A). Among tetraploid individuals, the EEN genome was characterized by the lowest percentage of abnormal individuals at both 18 °C (14.0%) and 28 °C (0.0%) (Figure 5). A statistically significant increase in the number of abnormal individuals was observed in the progeny of females with the ETN genome (49.0%) compared to the EEN genome (13.0%) at 18 °C (*p* < 0.05). At 28 °C, a statistically significant increase in abnormal individuals was observed among the progeny of females with the EET (75.6%) and ETN (57.9%) genomes, compared to the EEN genome (0.0%) (Figure 4B).

### 2.4. Body Length of Progeny

Measurements were taken on the third day post hatching. Larvae originating from females with the EEN genome had body lengths ranging from 5.0 to 7.8 mm at 18 °C (mean 6.8 mm), from 6.2 to 7.9 mm at 22 °C (mean 7.3 mm) and from 7.9 to 8.4 mm at 28 °C (mean 8.1 mm). Larval lengths differed significantly among all tested temperatures in this genomic composition (*p* < 0.05).

In the EET genome, larval body length ranged from 5.3 to 7.3 mm (mean 6.6 mm) at 18 °C, from 5.1 to 7.3 mm (mean 6.5 mm) at 22 °C, and from 4.9 to 7.7 mm (mean 6.3 mm) at 28 °C, with no significant differences between temperatures (*p* > 0.05).

Larvae originating from females with the ETN genome measured between 5.0 and 7.9 mm (mean 6.6 mm) at 18 °C, between 4.8 and 7.2 mm (mean 6.6 mm) at 22 °C, and between 4.2 and 8.2 mm (mean 6.8 mm) at 28 °C. Again, there were no significant differences between temperatures (*p* > 0.05).

A comparative analysis revealed that, at 22 °C, the larval body length of the EEN genomic composition was significantly greater than that of the EET and ETN compositions (*p* < 0.05). However, at 28 °C, significant differences were observed among all genomic compositions (*p* < 0.05) (see Figure 5).

## 3. Discussion

This research contributes to a broader understanding of how environmental factors, particularly water temperature, influence fish reproduction and early development. The study is especially relevant in the context of progressively rising water temperatures, a trend observed worldwide since the late 19th century [34]. Variations in temperature have been shown to affect not only the rate of embryonic and larval development, but also growth efficiency and survival in many fish species [36,37,38,39]. By focusing on triploid hybrid females, which dominate *Cobitis* populations in inland waters across Central Europe [19,21], the present study provides an important model for examining how genomic architecture interacts with environmental temperature to shape reproductive potential and developmental stability in polyploid fishes.

This study demonstrates that both the genomic composition of triploid *Cobitis* females and the temperature of egg incubation determine the early developmental success of their offspring. A consistent relationship between the quantity and quality of progeny was observed across all three genomic groups (EEN, EET, ETN). Offspring of EET and ETN females were characterized by higher hatching success but also by a greater frequency of developmental abnormalities, whereas progeny of EEN females showed lower hatching success combined with fewer abnormalities and greater body length during the first days after hatching. Temperature heterogeneously influences these outcomes. The intermediate temperature of 22 °C supported the most favorable developmental performance and the lowest incidence of deformities, while both lower (18 °C) and higher (28 °C) temperatures increased the proportion of malformed larvae, particularly among EET and ETN progeny. In contrast, offspring of EEN females were comparatively resistant to temperature variation, exhibiting consistently low deformity rates and a clear positive dependence of body length on temperature (28 °C > 22 °C > 18 °C at 3 dph).

### 3.1. Female Genome Structure

In natural diploid–polyploid *Cobitis* populations in Poland, triploid females can make up to 95% of the population. The remainder of the fraction consists of diploid *C. taenia* (TT), *C. elongatoides* (EE), their diploid hybrids (which sometimes may dominate locally; [21]), and small number of allotetraploids of both sexes [29].

In this study, we induced the reproduction of triploid females of hybrid origin, whose genomic composition indicated ancestry from two (EEN, EET) or three (ETN) different *Cobitis* species. The analyzed females were assigned to three distinct genomic groups: EEN, EET and ETN, with corresponding karyotypes of 3n = 75, 3n = 74* and 3n = 74, respectively. The genome composition of the analyzed females was reliably determined through the combined karyotype and microsatellite analyzes, an approach previously demonstrated to provide high accuracy in *Cobitis* hybrids [14,19,20].

### 3.2. Hatching Success

The hatching success of offspring differed markedly among genomic groups. The mean hatching success of triploid *Cobitis* females ranged between approximately 10% in EEN, 31% in EET, and 32% in ETN, with overall success rates declining at the highest temperature (28 °C). Although these values are lower than those reported for many teleost hybrids, they are broadly comparable to previous findings in *Cobitis* [40], where the mean hatching rate of allotriploid females ranged from 20 to 35% under similar experimental conditions. Such consistency indicates that the relatively low reproductive efficiency of triploid *Cobitis* reflects inherent genomic and developmental constraints rather than experimental conditions.

However, larvae of EEN origin—both triploid and tetraploid—displayed markedly fewer developmental abnormalities across all temperatures, and at 22 °C they exhibited the lowest proportion of abnormalities relative to EET and ETN offspring. Thus, despite producing fewer hatchlings, EEN females yielded higher-quality progeny, consistent with a strategy that prioritizes offspring viability over quantity. Such an adaptive strategy may enhance larval survival under natural conditions despite reduced reproductive output and may help explain why the oldest triploid clone, EEN, maintains higher phenotypic performance and fertility compared with the younger triploid forms, EET and ETN [41].

The present findings are consistent with earlier experimental data in *Cobitis* [40], where crosses between allotriploid females and diploid *C. taenia* males produced offspring with hatching success and 14-day survival comparable to those of diploid *C. taenia* and diploid *C. taenia* crosses, but with a higher incidence of skeletal deformities. Importantly, that study examined only differences between ploidy levels and did not assess the influence of specific genome composition. Our results extend this evidence by demonstrating that the genomic configuration (EEN, EET, ETN)—not only ploidy—determines the balance between hatching success and progeny quality in *Cobitis*.

Comparative data from other fish taxa provide broader context for interpreting these results. In hybrids of closely related species, reproductive success is often high despite genomic divergence. Crosses between white crucian carp (*Carassius cuvieri*) and red crucian carp *C. auratus* achieved hatching rates of around 80% [42], while hybrids produced between the Russian sturgeon (*Acipenser gueldenstaedtii*) and American paddlefish (*Polyodon spathula*), the so-called *sturddlefish*, exhibited remarkably high fertilization (86–93%) and hatching success (78–85%), with many larvae developing into viable triploids or pentaploids [43]. Despite their complex genomic background, the early survival of these interfamily hybrids remained relatively high (62–74%), illustrating that polyploidization can sometimes facilitate the success of otherwise improbable distant crosses.

In contrast, other hybrid combinations show little or no reduction in early developmental performance. For example, in Far Eastern dace (*Tribolodon*), F_1_ hybrids hatched at rates (~76%) comparable to those of purebred offspring (~86%), indicating that moderate genomic divergence does not necessarily impair embryo viability [44]. However, polyploidization often introduces developmental costs, even when hatching success is maintained. In crucian carp × zebrafish crosses, hatching rates were moderate, but hybrid larvae exhibited altered hatching enzyme activity and chorion properties associated with higher abnormality rates [45]. Likewise, induction of gynogenetic tetraploidy in Japanese flounder (*Paralichthys olivaceus*) led to a sharp decrease in hatching success (~47% compared with ~90% in controls) and an increase in deformities, confirming that additional genome duplication imposes significant developmental constraints [46].

Taken together, these examples demonstrate that hybridization and polyploidization exert variable effects on reproductive success. In some taxa, genome duplication reduces embryonic viability but enhances post-hatch robustness; in others, hatch rates remain stable but malformation rates increase; and in rare cases, even distant crosses can yield unexpectedly viable hybrids. Our results suggest that, in *Cobitis*, reproductive potential depends not only on ploidy level but also on the specific genomic composition of triploid females. The EEN lineage appears to follow a “quality-over-quantity” strategy, producing fewer but more viable offspring, whereas EET and ETN clones achieve higher hatching success at the cost of increased malformations.

Mechanistic studies conducted so far in *Cobitis* and other cyprinids do not yet allow for a clear explanation why EEN, EET, and ETN differ in reproductive outcomes. In triploid hybrid *Cobitis*, transcriptomic analyses have shown that the duplicated genome often dominates gene expression, generating dosage-dependent phenotypic biases [15]. However, dosage asymmetry alone cannot explain our findings, because both EEN and EET share a duplicated *C. elongatoides* genome but produce offspring of contrasting quality. It is possible that the difference arises from interactions between the duplicated genome and the third, non-duplicated genome. The *N* subgenome (from *C. tanaitica*) appears to be more compatible with the duplicated *E* genome than the *T* subgenome (from *C. taenia*), although this idea is not strongly supported by existing data. Current phylogenetic and genetic studies (mtDNA and nuclear DNA) indicate a similar degree of relatedness between E-T and E-N pairs [27]. Cytogenetic studies reveal that E and N share greater karyotype similarity than E and T [27], although T and N share a similar rDNA distribution pattern [20]. Gene expression and regulatory element analyses to date have focused on the E-T pair. Bartoš et al. [15] found that E × T hybrids exhibit mostly “intermediate” gene expression, with a bias toward the *C. taenia* genome in somatic tissues. The dominance of the T subgenome in gene expression and the strong role of cis-regulatory variants in this pair suggest significant regulatory differences between E and T. On the other hand, there are no publications on expression analysis or cis-regulatory sequences directly comparing *C. elongatoides* and *C. tanaitica*. It cannot be excluded that transcriptional homeostasis and dosage compensation between *E* and *N* subgenomes in EEN females may be more stable, thereby reducing regulatory interference during oogenesis and early embryogenesis. In contrast, the *T* subgenome in EET hybrids may show greater divergence in promoter structure, repetitive element content, and heterochromatin organization, potentially increasing transcriptional noise and asynchronous gene expression during development.

Research in other cyprinids may be helpful to this interpretation. Triploid and allopolyploid fishes exhibit asymmetric gene expression, partial dosage compensation, and pronounced maternal effects that influence developmental stability [47,48]. Transcriptome-wide analyses demonstrate that distinct genomic combinations produce highly variable expression profiles of homeologous genes, even when one genome is duplicated [49]. At the chromosomal level, studies of amphitriploid fish suggest that structural interactions between subgenomes are key determinants of developmental stability and evolutionary persistence [50]. In cyprinid allotetraploids, subgenomes initially evolve asymmetrically but gradually achieve a more balanced transcriptional state during rediploidization [51]. Recent studies further suggest that maternal subgenome dominance may be influenced by interactions between nuclear and mitochondrial genomes and by the distribution of transposable elements [52]. While conclusive evidence is lacking, it is plausible that variation in subgenomic compatibility and transcriptional coordination contributes to the divergent developmental outcomes among EEN, EET, and ETN hybrids. Further research is needed in this area.

The present study demonstrated a significant effect of incubation temperature on the development and hatching success of polyploid *Cobitis* larvae. Temperature is one of the key abiotic factors controlling proper embryogenesis in fish. Each species has a specific temperature range (thermal window) within which viable larvae can hatch successfully [53,54]. In our experiments, incubation across a wide temperature range (18–28 °C) resulted in successful offspring production. Both lower (18 °C) and higher (28 °C) temperatures increased the frequency of deformities, particularly among EET and ETN offspring. The lowest incidence of abnormalities across all genomic groups was observed at 22 °C. The observed heterogeneity in the effects of temperature on the reproductive success of hybrid *Cobitis* females also reflects inter-female variability. These females, being highly environmentally flexible, are likely capable of rapid adaptation to new thermal conditions.

Fish eggs are highly sensitive to ambient temperature; increased temperatures lead to shorter incubation periods [55]. Loaches of the genus *Cobitis* are known to reproduce within a temperature range of approximately 18–28 °C [35]. In our study, despite identical fertilization times, the duration of embryo incubation differed markedly: hatching occurred after three days at 28 °C, four days at 22 °C, and five days at 18 °C. Similar relationships between an increase in water temperature and a higher frequency of larval deformities have been reported in other fish species, including Atlantic cod (*Gadus morhua*; [56]), common carp (*Cyprinus carpio*; [57]), Senegalese sole (*Solea senegalensis*) [58]) and zebrafish (*Danio rerio*) [59]). Moreover, temperature-dependent effects on hatching and larval development have been documented in several hybrid and polyploid fishes. In African catfish hybrids (female *Clarias gariepinus* × male *C. macrocephalus*), elevated incubation temperatures accelerated yolk-sac resorption but reduced larval viability due to desynchronization between metabolic and morphological development [60]. In channel × blue catfish hybrids (female *Ictalurus punctatus* × male *I. furcatus*), temperature was identified as a primary influence on hatching success and early survival, with intermediate regimes being most favorable [61]. Similarly, in hybrid groupers (female *Epinephelus fuscoguttatus* × male *E. polyphekadion*), water temperature higher than the optimum range resulted in a sharp increase in larval abnormalities and a remarkable decrease in hatching rates [62]. Hybrid pufferfish (female *Takifugu obscurus* × male *T. rubripes*) grew and survived best at moderate temperatures, while both lower and higher temperatures impaired embryogenesis and increased mortality [63]. Both our and previous studies highlight a universal pattern among teleosts: while moderate warming can promote faster embryogenesis, further temperature increases push developmental systems beyond their physiological limits, leading to decreased survival and higher rates of abnormalities.

The temperature optimum revealed in the present study, between 18 °C and 28 °C, is also ecologically realistic for *Cobitis* reproduction. Field observations from Polish populations of *C. taenia* and *C. elongatoides* indicate that spawning takes place when the water temperature is above 16–18 °C and continues from May to July, when shallow marginal habitats usually range from 19 to 25 °C [25]. Spined loaches exhibit portion spawning and actively select warmer microhabitats, often several degrees above the ambient temperature, that are 20–26 °C [64,65]. Since this fish reproduces in shallow areas where water heats rapidly, spawning at about 22 °C closely matches natural conditions during the breeding season in Central Europe. However, progressive climate warming and the increasing frequency of extreme heat events may periodically push temperatures in these habitats beyond the optimal thermal window, to which hybrid *Cobitis* females are adapted. Water temperatures near 28 °C, shown here to cause increased mortality and deformities, are already recorded in Polish rivers and lakes during summer heatwaves. Rising temperatures are also predicted to lower dissolved oxygen levels [66], further threatening embryo viability. Broader evidence supports these ecological implications: temperature, more than flow, predicts reproductive timing in fishes [67]; spawning phenology is shifting earlier with warming [68]; and endocrine regulation along the brain–pituitary–gonad axis is highly temperature-sensitive [69,70,71]. Thus, while moderate warming may initially extend spawning opportunities, recurring heatwaves and oxygen depletion will likely reduce recruitment success and long-term population stability [72].

### 3.3. Ploidy and Phenotype of Progeny

Analysis of ploidy revealed no significant differences in the proportion of triploid and tetraploid progeny among genomic groups or incubation temperatures, a pattern consistent with earlier observations in *Cobitis* [29]. In our experiment, only male *C. taenia* served as sperm donors. When fertilization occurred, incorporation of paternal genetic material resulted in tetraploid offspring of both sexes [21,73], whereas during gynogenesis, sperm only activated embryogenesis without genetic contribution, leading to clonal triploid offspring. This dual reproductive mode—sexual and gynogenetic—is characteristic for *Cobitis* hybrids and represents an intermediate stage of hybrid speciation, where premeiotic endoreplication compensates for meiotic incompatibility between parental genomes [26]. Similar reproductive strategies have been described in other cyprinids, such as *Carassius gibelio* and *Misgurnus* loaches [74,75,76,77]. In both the present and earlier studies [29], triploid and tetraploid larvae initially occurred in similar proportions, but the frequency of tetraploids declined during post-hatching development, approaching ratios observed in natural populations. This pattern suggests that tetraploids exhibit higher developmental mortality, which corresponds with their increased incidence of morphological abnormalities (43.0% vs. 25.2% in triploids). Similar effects have been documented in other teleosts. Induction of gynogenetic tetraploidy in Japanese flounder (*Paralichthys olivaceus*) reduced hatching success (~47% compared to ~90% in controls) and increased deformity frequency, confirming the developmental costs of polyploidization [46].

In *Cobitis*, the frequency and type of deformities among tetraploids depended on the maternal genomic composition. Among tetraploid offspring, those with the EETT genome showed the highest proportion of abnormalities, ENTT were intermediate, and EENT the lowest, with differences most pronounced at 28 °C. This gradient indicates that simply doubling the *C. taenia* genome amplifies latent genomic incompatibilities, leading to reduced developmental stability at higher ploidy levels.

Mechanistically, these outcomes likely stem from processes previously discussed for *Cobitis*, including dosage imbalance, irregular chromosome pairing, incomplete rediploidization, rDNA instability, and variable in the efficiency of premeiotic genome duplication [15,20,28,78]. The genomic composition of tetraploids further influences the interactions among these mechanisms. Fertilization of triploid eggs by *C. taenia* males results in the formation of tetraploids with diverse combinations of *C. elongatoides*, *C. taenia*, and *C. tanaitica* genomes. Lines such as EENT, despite comprising three different copies of the *C. elongatoides*, *C. tanaitica*, and *C. taenia* genomes, appear to maintain more compatible regulatory interactions and, consequently, exhibit greater developmental stability. In contrast, EETT lines, which contain duplicate genomes from two species differing in chromosome number and regulatory architecture, may display a higher incidence of developmental defects in these tetraploids.

The high incidence of abnormalities in tetraploids, particularly those with less compatible genomic combinations, likely limits their viability in natural populations. Most tetraploid individuals represent transient or ephemeral lineages, while the more stable EENT configuration may occasionally persist long enough to act as a genetic bridge between clonal triploid forms. Nevertheless, the overall contribution of tetraploids to gene flow and recruitment appears minimal. This pronounced asymmetry in developmental success between triploid and tetraploid offspring may help explain why gynogenetic reproduction dominates in natural *Cobitis* populations, ensuring the long-term persistence of triploid clonal lineages under diverse environmental conditions.

### 3.4. Body Length of Progeny

In addition to differences in hatching success and developmental stability, our results showed that both incubation temperature and maternal genomic composition influenced the early growth of *Cobitis* larvae. Temperature exerts a major control over fish growth and body size, although its effects vary depending on species, life stage, and ecological context. In general, lower temperatures tend to produce larger size at hatching [79,80] because embryogenesis proceeds more slowly and yolk resources are utilized more completely, whereas higher temperatures accelerate metabolic rate and promote faster post-hatch growth within the species’ thermal tolerance [81,82]. In the present study, larval length was measured three days post-hatching, reflecting early post-hatch growth rather than size at emergence.

Against this background, offspring of EEN females exhibited a clear positive temperature response (28 °C > 22 °C > 18 °C), showing enhanced early growth under warmer conditions. In contrast, temperature had little effect on the body length of EET and ETN offspring, suggesting either a narrower thermal growth window or a physiological trade-off in which energy was diverted from somatic growth toward processes maintaining homeostasis during thermal stress.

Similar relationships between early thermal environment and larval growth have been described in other fish species. In hybrid yellow catfish (female *Tachysurus fulvidraco* × male *Pseudobagrus vachellii*), temperature and dissolved oxygen jointly shaped growth, survival, and oxidative capacity of newly hatched larvae [83], indicating that benefits of faster growth at higher temperatures are constrained by oxygen availability and metabolic capacity. Furthermore, in polyploid sterlet (*Acipenser ruthenus*), departures from the optimal thermal range reduced larval survival and condition, indicating that polyploid fishes may be particularly sensitive to suboptimal temperature regimes [84].

Our previous studies [40] showed that the average total length of progeny from triploid *Cobitis* females was statistically higher than that of diploid *C. taenia* offspring during the first ten days after hatching, although these differences disappeared after two weeks. This pattern is consistent with the well-known principle that elevated water temperature promotes faster initial growth but results in smaller adult body size [85]. Similar inverse relationships between temperature and growth have been observed in other teleosts: larval walleye pollock (*Gadus chalcogrammus*) exhibited growth rates inversely proportional to water temperature [86,87], and the developmental period of northern pike (*Esox lucius*) was approximately three times longer at the lowest tested temperature than at the highest [88].

Taken together, these observations agree with our results and indicate that temperature promotes early post-hatch growth only within a limited thermal window, beyond which developmental or metabolic constraints offset potential benefits. In our study, EEN larvae attained greater length at higher temperatures, consistent with accelerated growth within a favorable thermal range, whereas EET and ETN larvae showed little variation across temperatures, reflecting reduced thermal plasticity and tighter physiological regulation.

## 4. Materials and Methods

Twenty triploid *Cobitis* females, representing three different genomic compositions (EEN, EET and ETN) depending on the species with which they were hybridized, were crossed with diploid *C. taenia* males. Diploid males of *C. taenia* used as sperm donors came from two populations consisting exclusively of diploid individuals, all had 2n = 48 chromosomes. Their eggs and larvae were then incubated under three temperature conditions (18 °C, 22 °C and 28 °C) for three days after hatching.

### 4.1. Fish Sampling and Genomic Identification

The fish used in the crossing experiment were caught in June, which coincided with their natural spawning period, and the experiment was conducted over three consecutive years (2022–2024). Twenty allotriploid (3n) *Cobitis* females were collected from two diploid-polyploid populations: the Pilica River (51°34′29.6″ N, 20°20′16″ E) in the Vistula River drainage basin of the Baltic Sea, and twenty diploid (2n) *C. taenia* males were obtained from an exclusively diploid population in Lake Legińskie (53°58′40.9″ N, 21°08′28.0″ E) in the Pregoła River basin of the Baltic Sea drainage system. Mature males were identified by the presence of a *lamina circularis* at the base of the pectoral fins [25].

The ploidy levels of the parental individuals were determined before induced spawning by flow cytometry using fin clips, as previously described [89], and this was confirmed post-experimentally via chromosome counts. The genome of the 3n females was analyzed using species-specific microsatellite markers, sequencing of the nuclear S7 gene intron and karyotyping after crossing experiments, as previously described by Boroń et al. [20]. DNA was extracted from the dorsal fin or muscle tissue using a Genomic Mini Kit (A&A Biotechnology, Gdansk, Poland). Microsatellite genotyping was performed using an ABI PRISM 3130 Genetic Analyzer (Applied Biosystems, Waltham, MA, USA), with allele scoring performed with GeneMapper v3.7 (Applied Biosystems, Waltham, MA, USA) [90]. The S7 intron was amplified according to the method of Janko et al. [19,21] and sequenced commercially (Genomed, Warsaw, Poland). Sequence alignments were carried out manually in BioEdit v.7.2.5 (Ibis Biosciences, Carlsbad, CA, USA).

Karyotyping was performed to confirm ploidy and genomes. Live fish were injected with 1 mL of 0.05% colchicine per 100 g of body weight. After 1.5 h, the fish were euthanized with an overdose of MS222 (100 mg/L). The kidney cells were then hypotonized in 0.075 M KCl for 50 min and fixed in methanol:acetic acid (3:1). Chromosome spreads were prepared using the splash method and stained with 4% Giemsa [20]. The chromosomes were examined using a Nikon Eclipse 80i microscope (Nikon Corporation, Tokyo, Japan). A minimum of 12 metaphase spreads per individual were analyzed using MultiScan v14.02 software (Computer Scanning Systems, Warsaw, Poland; multiscan.idsl.pl). The chromosomes were classified according to Levan et al. [91]. The karyotypes of triploid hybrid parental females were analyzed as previously described in detail by Boroń et al. [20].

### 4.2. Crossing Experiments

**Figure 6:**
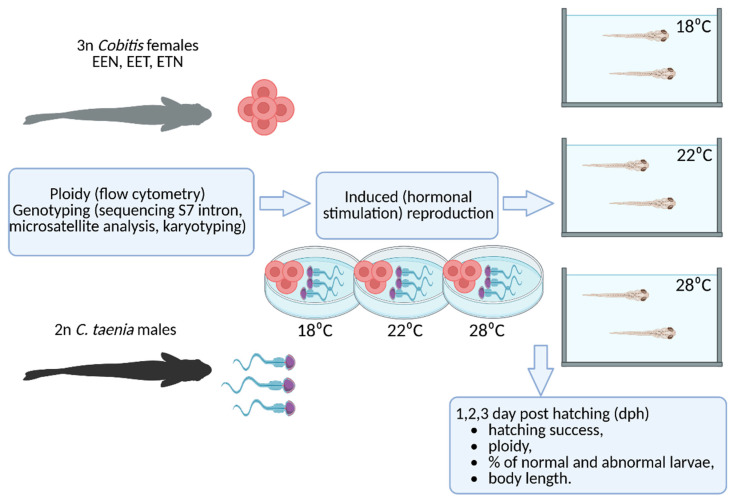
Experimental design scheme (created with BioRender.com). *Cobitis* females were examined for ploidy using flow cytometry. Genotyping was performed using S7 intron sequencing, microsatellite analysis, and karyotyping. Crossing experiments were conducted between triploid *Cobitis* females with three different genomes (EEN, EET, and ETN) and diploid *C. taenia* males. Artificial spawning and subsequent larval development took place at three temperatures: 18 °C, 22 °C, and 28 °C. The offspring obtained were examined for hatching success, ploidy, percentage of abnormalities, and body length in the first three days after hatching.

Hormonal stimulation of the parent fish was carried out using Ovopel (a GnRH analog and metoclopramide) (Interfish Ltd. Budapest, Hungary), as described by Juchno et al. [29]. The eggs collected from each triploid female were divided into three portions and fertilized with sperm from *C. taenia* in Petri dishes at the following temperatures: 18 °C, 22 °C and 28 °C. The fertilized eggs were then transferred to 50 L aquaria within closed water circulation systems, each of which was maintained at the corresponding temperature. The eggs were checked daily and any dead eggs, identified by whitening, were removed using a Pasteur pipette. The larvae were reared under controlled conditions with a light–dark cycle to mimic natural conditions. Subsets of the larvae were sampled at 1, 2 and 3 days post hatching (dph) for further analysis (see Figure 6). Offspring were examined during the first three days post-hatching since previous studies have demonstrated a rapid decline in the number of tetraploid offspring after this timeframe [29].

The following parameters were determined for larvae obtained from females with three genomes (EEN, EET and ETN) that were reared at three different temperatures:

### 4.3. Hatching Success

Hatching success was calculated as a percentage of the total number of eggs obtained, relative to the number of larvae hatched at 1 dph.

### 4.4. Ploidy Analysis of Progeny

The ploidy status of approximately 10 randomly selected offspring per each cross was determined by flow cytometry (Table 3), following the method of Jablonska et al. [89] with slight modifications. Larvae collected at 1, 2 and 3 dph were used for nuclei isolation. The nuclei were stained using the CyStain UV Precise T kit (Sysmex Partec GmbH, Görlitz, Saxony, Germany) and analyzed using a CyFlow Ploidy Analyzer (Partec GmbH, Görlitz, Saxony, Germany). The relative DNA content was then compared against a diploid reference from *C. taenia* × *C. taenia* progeny.

### 4.5. Phenotype Analysis of Progeny

To assess normal and abnormal development, the external morphology of approximately 10 randomly selected larvae from each cross was examined up to three days post hatching (dph) (Table 3). Larvae deformities were categorized according to the descriptions provided by Jezierska et al. [92] and Alix et al. [93]. We classified a larva as abnormal if it exhibited any developmental malformation. Representative images of normal and abnormal progeny across different ploidy levels and genomes were captured using NIS-Elements F 4.60.00 64-bit imaging software and a Nikon SMZ1270 microscope (Nikon Corporation, Tokyo, Japan).

### 4.6. Measurement of Body Length in Progeny

Larval body measurements were taken using NIS-Elements F imaging software to determine the standard length (SL in mm), which was measured from the tip of the snout to the end of the caudal peduncle. Approximately 10 individuals were randomly selected from each cross and measured at 3 dph.

### 4.7. Statistical Analysis

For statistical analysis, we pooled data from three years (spawning seasons) because the experimental conditions were identical in each year. Percentage data were arcsine-transformed prior to statistical analysis. The normality of the distribution was checked using the Shapiro–Wilk test. One-way ANOVA was performed using Statistica v13.3 software (TIBCO Software Inc., Palo Alto, CA, USA) to determine the effects of the 3n *Cobitis* female genome composition, water temperature, and ploidy distribution on hatching success and the external morphology of the larvae. Differences between the studied groups were assessed using the Tukey HSD post hoc test.

## 5. Conclusions

This study provides the first experimental evidence that early developmental success in *Cobitis* hybrids is shaped by genomic composition and incubation temperature, reflecting complex interactions between inherited genomic architecture and environmental conditions. The contrasting reproductive strategies of the triploid lineages—quantity-biased in EET and ETN, and quality-biased in EEN—illustrate how polyploid systems balance offspring number and viability in response to both genetic and thermal conditions. Furthermore, the elevated abnormality rates observed in tetraploids highlight the developmental costs of increased ploidy and help explain why triploid gynogenesis dominates in natural populations.

These findings provide a solid foundation for further research into the mechanisms underlying genome–environment interactions in polyploid fishes, particularly how female genomic composition and rising water temperatures influence reproductive success, developmental stability, and the evolution of *Cobitis* hybrid lineages. Understanding these processes will be essential for predicting how hybrid and clonal fish systems respond to ongoing climatic change and for identifying the genetic and ecological factors that support their long-term stability.

## Figures and Tables

**Table 1:** Number of eggs, hatched larvae, and hatching success (%) of offspring produced by allotriploid *Cobitis* females of different genome compositions (EEN, EET, and ETN) fertilized by diploid *C. taenia* males at three incubation temperatures (18 °C, 22 °C, and 28 °C). The table summarizes variation in reproductive performance according to female genome composition and incubation temperature.

Family	Number of Eggs			Number of Hatched Larvae			Hatching Success (%)		
	18 °C	22 °C	28 °C	18 °C	22 °C	28 °C	18 °C	22 °C	28 °C
Triploid *Cobitis* females of EEN genome composition									
F3M3	259	351	350	87	100	99	33.6	28.5	28.3
F35M35	243	224	186	96	4	0	39.5	1.8	0.0
F37M36	139	145	139	10	10	0	7.2	6.9	0.0
F39M37	187	175	237	7	2	0	3.7	1.1	0.0
F47M47	343	304	234	0	0	9	0.0	0.0	3.9
						Mean	16.8	7.7	6.4
Triploid *Cobitis* females of EET genome composition									
F67M55	268	388	447	63	188	8	23.5	48.5	1.8
F69M57	233	251	271	38	63	20	16.3	25.1	7.4
F70M58	213	322	212	10	133	31	4.7	41.3	14.6
F71M59	127	140	128	34	92	23	26.8	65.7	18.0
F72M60	168	199	208	142	136	61	84.5	68.3	29.3
						Mean	31.2	49.8	14.2
Triploid *Cobitis* females of ETN genome composition									
F5M5	167	123	128	77	39	0	46.1	31.7	0.0
F10M10	213	190	216	84	146	0	39.4	76.8	0.0
F11M11	192	272	314	55	4	130	28.7	1.5	41.4
F12M12	289	363	334	0	43	0	0.0	11.9	0.0
F46M46	169	186	197	26	35	23	15.4	18.8	11.7
F48M48	141	124	120	35	57	31	24.8	46.0	25.8
F57M52	199	253	219	137	51	3	68.8	20.2	1.4
F73M61	85	124	157	57	34	58	67.1	27.4	36.9
F75M63	106	94	92	37	38	6	34.9	40.4	6.5
F78M66	163	125	135	151	89	94	92.6	71.2	69.6
						Mean	41.8	34.6	19.3

Family’ refers to a pair of *Cobitis* individuals, one female (F) and one male (M) selected for producing offspring through induced spawning.

**Table 2:** Triploid (3n) and tetraploid (4n) progeny (in%) according to genome composition (EEN, EET, ETN) and water temperature (18 °C, 22 °C, 28 °C). It was observed that the mean proportions of triploid and tetraploid offspring were comparable across all genome compositions and temperatures, with no statistically significant differences detected.

		Ploidy	Range	Mean	±SE
	EEN	3n	0.0–52	37.6	6.0
		4n	20.0–55.6	41.6	4.5
Female	EET	3n	8.3–75.0	52.6	4.3
genome		4n	25.0–91.7	43.3	4.6
	ETN	3n	4.8–73.1	41.4	4.4
		4n	10.0–80.0	40.5	4.0
	18 °C	3n	0.0–72.2	45.5	4.8
		4n	16.7–80.0	39.2	4.4
Temperature	22 °C	3n	8.3–70.6	42.8	4.1
		4n	10.0–91.7	43.0	5.0
	28 °C	3n	0.0–75.0	40.1	7.7
		4n	17.1–100.0	48.3	6.7

**Table 3:** Sample size for ploidy and phenotype analysis according to genome composition (EEN, EET, ETN) and water temperature (18 °C, 22 °C, 28 °C).

Temperature	Female Genome	Number of Larvae
18 °C	EEN	51
	EET	45
	ETN	143
	Total	239
22 °C	EEN	38
	EET	53
	ETN	124
	Total	215
28 °C	EEN	23
	EET	62
	ETN	97
	Total	182

## Data Availability

The original contributions presented in the study are included in the article, further inquiries can be directed to the corresponding author.
